# Physical Wellbeing Scavenger Hunt

**DOI:** 10.5070/M5.53258

**Published:** 2026-10-31

**Authors:** Walker Plash, Grace Lagasse

**Affiliations:** * University of South Alabama, Department of Emergency Medicine, Mobile, AL

## Abstract

**Audience:**

This small group session is appropriate for any level of medical student, emergency medicine resident physicians, and faculty.

**Introduction:**

Burnout is a major issue in medicine, with over half of physicians experiencing burnout at some point in their career.^1^ Physicians who regularly participate in self-care activities, such as regularly exercising, having a well-balanced diet, and appropriate amount of sleep, experience less burnout.^2^,^3^,^4^ As a resident, it is difficult to find time to practice self-care.^2^ This session sought to combine education on how to implement appropriate self-care as a trainee and how to carry that forward after training in a way that was fun, provided a period of physical exercise, and provided time outdoors for those participating.

**Educational Objectives:**

By the end of this session, the learner will be able to: 1) implement basic ways to improve sleep quality, 2) avoid common pitfalls in diet and nutrition, 3) incorporate more physical activity into their lives.

**Educational Methods:**

Learners were divided into teams of three to four. A scavenger hunt was created using Google Forms and QR codes. The initial QR code was displayed in the lecture hall at the start of the session. Fifteen other QR codes were either physically distributed throughout the Emergency Medicine Offices and the Emergency Department or provided to participants via text message when they completed certain tasks. Each QR code led to a different Google Form with a question about physical wellbeing. Participants were allowed to use any resource they had with them, including their mobile devices. After the question was answered correctly, an explanation was provided in the Google Form. A riddle was also provided to help locate the next QR code. An incorrect answer required the participants to try to answer the question again. Participants were not given any hints after answering incorrectly. There were no formal penalties for incorrect answers. However, incorrect answers increased the time it took to complete the task. At the end of the session, each question was debriefed by the instructor.

**Research Methods:**

Participants completed a five-question pre-survey to rate their level of physical wellbeing using a 1–5 Likert Scale. Participants also completed a 10-question post survey that asked them the same initial 5 questions to rate their physical wellbeing on the same scale as the pretest, as well as additional questions asking how likely they were to change their physical habits based on the session and how much they enjoyed the session on a different 1–5 Likert Scale. They were also asked to provide recommendations for future changes. Pre- and post-test Likert scores were compared using the Mann-Whitney U Test.

**Results:**

Twelve participants, all resident physicians, took part in the exercise. Twelve completed the pre-survey, and ten completed the post-survey. There was no difference in physical wellbeing score (median 3 before vs median 2.5 after, *P*=0.62). All participants except one were at least moderately likely (3 or above on a 1–5 Likert scale) to change their sleep habits based on the exercise. All were at least moderately likely (3 or above on a 1–5 Likert scale) to change their eating and exercise habits based on the activity. All respondents found the activity to be at least moderately enjoyable (3 or above), with 8 of 10 finding it extremely enjoyable (5).

**Discussion:**

This wellbeing scavenger hunt effectively taught learners how to incorporate simple changes into their lives to improve the self-care habits. This model could be applied to other educational topics to incorporate a component of physical wellbeing and exercise and has been used during new resident orientation and wilderness education within our department.

**Topics:**

Wellbeing, physical wellbeing, exercise, nutrition, sleep habits, self-care.

## USER GUIDE

**Table:** 

List of Resources:	
Abstract	1
User Guide	3
Small Groups Learning Materials	6
Pearls	22

**Learner Audience:**

Any level of medical student, emergency medicine resident physicians, and faculty

**Time Required for Implementation:**

Approximately two hours: thirty minutes for setup, one hour for the activity, and thirty minutes for the debrief.

**Recommended Number of Learners Per Instructor:**

There is no strict limit because the activity is self-guided and debrief can be performed in a large group format. However, it is recommended that 3–5 participants form a team with a maximum of 30 participants per instructor to ensure a meaningful debrief.

**Topics:**

Wellbeing, physical wellbeing, exercise, nutrition, sleep habits, self-care.

**Objectives:**

By the end of this session, the learner will be able to:

1. Implement basic ways to improve sleep quality
2. Avoid common pitfalls in diet and nutrition
3. Learn to incorporate more physical activity into their lives.

### Linked objectives and methods

The goal of this session was to educate learners on ways to improve their physical wellbeing and self-care habits in a fun and interactive session. Gamification with a competitive scavenger hunt was used as a tool to increase learner engagement. The scavenger hunt component required learners to participate in a physical activity component, thus reinforcing how to incorporate physical activity into their day. The virtual component, including questions at each stage, allowed for educational material to be interspersed throughout the activity without requiring an instructor at each station. Participants were allowed to create their own team as long as there was representation across all levels of learners participating in the activity.

### Recommended pre-reading for facilitator

Pre-reading allows facilitator to guide the debrief. Each reading is approximately two pages in length and the total time to review should not take more than 20 minutes. Faculty can complete the prereading while the scavenger hunt is ongoing prior to the debrief if needed.

- American Heart Association. American Heart Association Recommendations for Physical Activity in Adults and Kids. American Heart Association. Published January 19, 2024. Accessed September 24, 2025. https://www.heart.org/en/healthy-living/fitness/fitness-basics/aha-recs-for-physical-activity-in-adults
- Circadian adaptation to shift work. Developed by the American Academy of Sleep Medicine. Accessed September 25, 2025. https://aasm.org/wp-content/uploads/2022/07/ProviderFS-ShiftWork.pdf
- Newsom R. Sleep Debt: Can you catch up on sleep? Sleep Foundation. Published April 1, 2022. Accessed September 25, 2025. https://www.sleepfoundation.org/how-sleep-works/sleep-debt-and-catch-up-sleep
- Caffeine, food, alcohol, smoking and sleep. Sleep Health Foundation. Accessed September 23, 2025. https://www.sleephealthfoundation.org.au/sleep-topics/caffeine-food-alcohol-smoking-and-sleep
- Healthy eating. University of Michigan Health. Uofmhealth.org. Published 2024. Accessed September 25, 2025. https://www.uofmhealth.org/health-library/nutri#aco4367

### Learner responsible content (LRC)

Learners were encouraged to use their mobile devices in real time to look up answers from variable sources including but not limited to the facilitator pre-reading references to assist in the team discussion.

### Results and Tips for Successful Implementation

This activity is best implemented in a combined session with multiple levels of learners at one time. Ideally, it is implemented in an area with room to hide QR codes a distance apart to encourage physical activity with time to be outdoors as part of the activity. This activity requires a big enough classroom to accommodate the number of participants involved as well as pre-designated areas outside of the classroom. The areas outside of the classroom should ideally be a minimum of 100 meters away from the classroom to ensure adequate physical exertion during participation. This session was implemented on May 31, 2023, as the last hour of the regularly scheduled educational conference. It was implemented in and around the Department of Emergency Medicine offices and the Emergency Department, which is approximately 200 meters away. Twelve learners, all emergency medicine (EM) residents in post-graduate years 1–3, participated in person. This activity is performed as part of a three-year rotating wellbeing curriculum and will be performed during 2026 academic year.

We created a five-question pre-test that asked for what level of training the participant was in, as well as to rate their level of overall physical wellbeing, sleep quality, diet quality, and exercise frequency on a Likert scale of 1 (worst) to 5 (best). Afterwards, participants completed a 10-question post-test that asked the same initial five questions on the same scale, as well as how likely they are to change their sleep habits, exercise habits, and diet habits on a 1 (not at all) to 5 (extremely likely) Likert scale. They were also asked how much they enjoyed the activity on a 1 (not at all) to 5 (excellent) Likert scale and to provide any formative feedback as free text.

Data are expressed as median values and interquartile ranges. The Mann-Whitney U test was used to analyze scores of items on both the pre- and post-test. This test was used because there were an uneven number of participants who completed the pre- and post-test, so a paired test was not feasible. Additionally, given the small sample size, using dummy values for the two missing participants was felt to have a high likelihood of influencing results.

Twelve participants completed the pretest (3 PGY-1s, 5 PGY-2s, 3 PGY-3s, one did not answer). Ten participants completed the post-test (3 PGY-1s, 4 PGY-2s, 3 PGY-3s. There was no difference in overall physical wellbeing (3, IQR 2–3 vs 2.5, IQR 2–3; *P*=0.62), sleep quality (3, IQR 2–3 vs 3, IQR 2.25–3; *P*=0.84), diet quality (3, IQR 2–3 vs 2.5, IQR 2–3; *P*=0.74), and exercise frequency (2, IQR 1.75–3 vs 2, IQR 1.25–2.75; *P*=0.84). Additionally, the percentages of participants with scores above three were comparable for all (overall physical wellbeing 0% before vs 0% after, sleep quality 17% before vs 20% after, diet quality 0% before vs 0% after, exercise frequency 8% before vs 10% after). All respondents on the post survey except one were moderately likely (3) or greater to change their sleep habits based on the exercise (median=3, IQR 3–4). All respondents were moderately likely (3) or greater to change their eating habits (median=3, IQR 3–4) and exercise habits (median=3.5, IQR 3–4). All respondents found the activity to be at least moderately enjoyable (3), with 8 of 10 finding it extremely enjoyable (5) (median=5, IQR 5–5).

The only change recommended in the post session comments was to warn nursing and ancillary staff in the emergency department so as to not alarm them when residents are moving hastily through the department. Future iterations of this activity will incorporate this feedback as well as be trialed on larger groups of learners.

Some of the riddles to guide to the next clue will not be applicable to all educators because they were specific to our institution. These riddles that will need to be modified are highlighted in green in the sGAE. Those implementing this material can use different riddles or simply provide instructions on where to go for the next QR code.

## SMALL GROUPS LEARNING MATERIALS

### Small Group Application Exercise (sGAE)

Before the activity, printed QR codes leading to Google Forms should be placed throughout the emergency department. They may also be placed in nearby areas, such as administrative offices. Each location should have only one QR code, and the QR codes should be far enough apart so that the learners will achieve moderate exercise during the activity. Learners should start in a classroom where they will be provided instructions for the activity. Learners should be instructed that they will need their mobile devices for the activity to scan QR codes and can use any electronic resource they would like. They should also be instructed that each QR code will have a question or task, which they must complete correctly. They will then receive the riddle to lead them to the next QR code. Since the answers to the riddles are a location, some locations are specific to our health system. Riddles that may need to be reworked or replaced, or just replaced with the name of the location, are highlighted in green in the sGAE. The first team that returns to the classroom will win the activity. The moderator should stay in the classroom to identify the winning team, and other faculty should walk through the course to address any issues in real time. To start the activity, the first QR code should be displayed on the screen in the classroom.

### Small group application exercise (sGAE) Questions

Question 1:

Which of these is an example of a Zeitgeber?

- Ambien
- Sleep hygiene
- Sunlight
- Thomas Schedule

Next Clue:

Arthur and his knights here would be proud to treat, because at this table there is no head seat. So, pack up and go get your mail, for there is the next stop on this trail.

Question 2:

What is anchor sleep?

- Listening to specific frequencies of ambient sound while sleeping
- Maintaining a 4-hour block of sleep within the same time in a 24-hour period
- Sleeping for short periods throughout the day instead of one long session at night
- Use of weighted blankets infused with calming essential oils

Next clue:

Traveling up is the way to advance. But know that the journey is part of the dance. If you skip a floor on the way, you may miss the answer today. So use your legs on the way to the top, and there you will find the next place to stop.

Question 3:

Which of these is unlikely to improve sleep after a night shift?

- Blackout curtains
- Consuming alcohol
- Limiting caffeine prior to sleep
- Limiting screen time prior to sleep

Next clue:

To receive the next link, one member of the group should text a selfie with their child or pet to the moderator for the session. The moderator then texts back the next link upon receiving it.

Question 4:

Is it easier to transition your schedule clockwise or counter-clockwise?

- Clockwise
- Counter-clockwise

Next clue:

The true leader of the residency stands at their desk, planning the leadership’s next quest. Look in their office as they keep the program afloat; there you will find the next question of note.

Question 5:

From a sleep standpoint, when is the best time to exercise?

- Immediately after a heavy meal
- Shortly after waking up
- Right before going to bed
- When running a fever

Next clue:

For the next question, two of the group must have had a primary care physician or dentist appointment within the past year. If not, two of the group must call and make an appointment for one or the other. Notify the moderator when this is complete, and they will release the next question.

Question 6:

What are the AHA recommendations for the amount of moderate exercise an adult should get per week?

- 75 minutes
- 120 minutes
- 150 minutes
- 180 minutes
- 300 minutes

Next clue:

In this hall the signs say “no cots”, but we all know they are stored here in lots. Walking into work, you’ll pass cracked concrete; take this door if you don’t mind stairs in the heat.

Question 7:

Which of these is an example of moderate intensity exercise, per the AHA?

- Aerobic dancing
- Gardening
- Hiking
- Running
- Swimming laps
- Tennis-singles

Next clue:

In a room filled with doctors charting away, some of you after your shifts too long do stay. Back-to-back, we all sit, while residents fill the air with wit. In the room where you learn things anew, search here for your next clue.

Question 8:

Brainstorm at least five ways members of your group can increase their amount of physical activity over the next six months.

Next clue:

For the next question, send a picture to the moderator of one member of the group outside doing a form of physical activity from the past six months. It could even be today!

The moderator then texts back the next link upon receiving it.

Question 9:/

How many hours of sleep does the average adult need per night?

- 4
- 5
- 6
- 7
- 8

Next clue:

The code to this room is an important date, and you may even be able to fill a plate. Only one restroom--enough that can’t be! So instead, here the designers built more restrooms--three!

Question 10:

Which of these is associated with improved healthy eating, especially when starting to change eating habits?

- Adding to your diet instead of taking away
- Crash dieting
- Diet pills
- Eliminating specific food groups
- Juice cleanses

Next clue:

Use me to see that without which you cannot. You are powerless to change where I look, only where I am.

Question 11:

Which of the options below may increase your risk of drowsy driving after a night shift?

- Driving faster to get home sooner
- Napping for 15 minutes prior to driving home
- Pulling over and taking a brief nap
- Taking public transportation
- Taking the hospital’s Free Taxi Service

Next clue:

Out in the woods, I heard such a clatter. I ran to my friend to see what was the matter. His arm bent in a way I had never seen, and I knew what that must mean. With duct tape and two sticks, his arm could be snared, or maybe use this, had I been more prepared. The man who created this couldn’t be dumb. He came up with the idea while studying a wrapper of gum.

Text the answer to the moderator. The moderator then texts back the next link upon receiving it.

Question 12:

Which of these may worsen your health?

- Decreasing sugar
- Increasing artificial sweetener
- Replacing saturated fats with unsaturated fats
- Increasing fiber
- Limiting processed foods

Next clue:

When fluid drains oh so foul, and the way of the kangaroo you have no time for now, use me to stent open the flow, and to the OB/GYN they will go. Fill me up with an 18-gauge needle, otherwise the flow of pus will be feeble. But not too much, the balloon will explode. Find me for the next QR code.

Question 13:

Which of these is unlikely to help you achieve your physical wellbeing goals?

- Set both short- and long-term goals
- Set goals based on your priorities
- Use vague goals that are difficult to measure
- Work with a partner to hold each other accountable
- Write down your goals, both short- and long-term

Next clue:

I am strong when what should be strong is broken. You won’t find me in the FED, but I am your only option at UH. Bro doctors say they can’t live without me.

Question 14:

What is one thing each group member will do to improve their quality of sleep? Each group member should have a different answer.

Next clue:

Here you will find the question for your victory to clinch, the hallway that leads to plastic for a pinch. From here candy abounds, and leaders for the department make their rounds.

Question 15:

Please list two changes that each of you can make in your food choices to improve your overall health. Each team member should list two separate changes.

Next clue:

Congratulations, you’ve reached the end of our game. Now back to the classroom for your prize to claim. The first team that makes it back with all answers complete wins a prize ever so sweet.

### Small group application exercise (sGAE) Answers

For all incorrect answers, residents receive the feedback “Incorrect, try again” and attempt the question again.

Question 1:

Which of these is an example of a Zeitgeber?

- Ambien
- Sleep hygiene
- Sunlight
- Thomas Schedule

Answer feedback:

Correct! Zeitgebers are environmental cues that synchronize our circadian rhythm. Sunlight is a significant one that triggers wakefulness. Wearing sunglasses on the way home after a night shift can help mitigate this, allowing improved sleep during the day between night shifts.

Next Clue:

Arthur and his knights here would be proud to treat, because at this table there is no head seat. So, pack up and go get your mail, for there is the next stop on this trail.

Answer- Resident Lounge

Question 2:

What is anchor sleep?

- Listening to specific frequencies of ambient sound while sleeping
- Maintaining a 4-hour block of sleep within the same time in a 24-hour period
- Sleeping for short periods throughout the day instead of one long session at night
- Use of weighted blankets infused with calming essential oils

Answer feedback:

Correct! Anchor sleep prevents you from switching off your regular circadian rhythm. For example, if you are working nights and normally sleep from 0800–1600 and have an off day with meetings, sleeping from 0400–1200 would give 4 hours of anchor sleep from 0800–1200. This would help prevent flipping your circadian rhythm. Anchor sleep is associated with more total sleep, reduced sleep debt, and shorter recovery time, especially if maintaining 0200–0600 as an anchor. This is difficult to do on 12-hour shifts.

Next clue:

Traveling up is the way to advance. But know that the journey is part of the dance. If you skip a floor on the way, you may miss the answer today. So use your legs on the way to the top, and there you will find the next place to stop.

Answer--stairwell

Question 3:

Which of these is unlikely to improve sleep after a night shift?

- Blackout curtains
- Consuming alcohol
- Limiting caffeine prior to sleep
- Limiting screen time prior to sleep

Answer feedback:

Correct! Although alcohol can help you to fall asleep more quickly, it changes the architecture of your sleep cycle, which can result in waking up in the middle of your sleep period or otherwise disturbed sleep. Alcohol proportionally decreases the amount of time spent in REM sleep and increases the time spent in Stage II sleep. All the others will help you sleep after a night shift.

Next clue:

To receive the next link, one member of the group should text a selfie with their child or pet to the moderator for the session. The moderator then texts back the next link upon receiving it.

Question 4:

Is it easier to transition your schedule clockwise or counter-clockwise?

Clockwise
Counter-clockwise

Answer feedback:

Correct! The natural human circadian rhythm is 25 hours. This makes transitioning to later shifts in the day (or traveling east to west) easier. Schedulers should work to maximize transitions in this direction and minimize transitions to earlier shifts.

Next clue:

The true leader of the residency stands at their desk, planning the leadership’s next quest. Look in their office as they keep the program afloat; there you will find the next question of note.

Answer--Residency program coordinator

Question 5:

From a sleep standpoint, when is the best time to exercise?

- Immediately after a heavy meal
- Shortly after waking up
- Right before going to bed
- When running a fever

Answer feedback:

Correct! Exercise increases wakefulness and can function as a cue to your circadian rhythm to start a wakeful cycle. This can be beneficial after waking up but can be harmful if trying to sleep soon.

Next clue:

For the next question, two of the group must have had a primary care physician or dentist appointment within the past year. If not, two of the group must call and make an appointment for one or the other. Notify the moderator when this is complete, and they will release the next question.

Question 6:

What are the AHA recommendations for the amount of moderate exercise an adult should get per week?

- 75 minutes
- 120 minutes
- 150 minutes
- 180 minutes
- 300 minutes

Answer feedback:

Correct! This can be broken up over any number of days.

Next clue:

In this hall the signs say no cots, but we all know they are stored here in lots. Walking into work you’ll pass cracked concrete; take this door if you don’t mind stairs in the heat.

Answer--back door hallway to the ED

Question 7:

Which of these is an example of moderate intensity exercise, per the AHA?

- Aerobic dancing
- Gardening
- Hiking
- Running
- Swimming laps
- Tennis-singles

Answer feedback:

Correct! All the other are considered vigorous. The AHA only recommends a minimum of 75 minutes a week of exercise if you are exercising vigorously.

Next clue:

In a room filled with doctors charting away, some of you after your shifts too long do stay. Back-to-back we all sit, while residents fill the air with wit. In the room where you learn things anew, search here for your next clue.

Answer--doctor workroom

Question 8:

Brainstorm at least five ways members of your group can increase their amount of physical activity over the next six months.

Answer feedback:

There are no incorrect answers! But some possibilities include always taking the stairs, parking as far away in the parking lot as possible, do a 5-minute circuit workout in the morning, and more.

Next clue:

For the next question, send a picture to the moderator of one member of the group outside doing a form of physical activity from the past six months. It could even be today! The moderator then texts back the next link upon receiving it.

Question 9:

How many hours of sleep does the average adult need per night?

- 4
- 5
- 6
- 7
- 8

Answer feedback:

Correct! 7 hours allows for the appropriate amount of each stage of sleep. Anything less generates a sleep debt, which causes worsening physical and mental performance. Interestingly, REM cycles increase in length the longer you sleep, so you are statistically more likely to wake up during a dream than not as you sleep longer.

Next clue:

The code to this room is an important date, and you may even be able to fill a plate. Only one restroom--enough that can’t be! So instead, here the designers built more restrooms--three!

Answer-staff breakroom

Question 10:

Which of these is associated with improved healthy eating, especially when starting to change eating habits?

Adding to your diet instead of taking away
Crash dieting
Diet pills
Eliminating specific food groups
Juice cleanses

Answer feedback:

Correct! Add foods that you think you need more of, like fruits and vegetables. If you start off by taking things out of your diet—like foods that are high in fat or sugar—you might feel deprived. And that will make it harder for you to change.

Next clue:

Use me to see that without which you cannot. You are powerless to change where I look, only where I am.

Answer--slit lamp

Question 11:

Which of the options below may increase your risk of drowsy driving after a night shift?

Driving faster to get home sooner
Napping for 15 minutes prior to driving home
Pulling over and taking a brief nap
Taking public transportation
Taking the hospital’s Free Taxi Service

Answer feedback:

Correct! All of the other options are effective way to address fatigued driving.

Next clue:

Out in the woods, I heard such a clatter. I ran to my friend to see what was the matter. His arm bent in a way I had never seen, and I knew what that must mean. With duct tape and two sticks, his arm could be snared, or maybe use this, had I been more prepared. The man who created this couldn’t be dumb. He came up with the idea while studying a wrapper of gum.

Text the answer to the moderator. The moderator then texts back the next link upon receiving it.

Answer--SAM splint

Question 12:

Which of these may worsen your health?

- Decreasing sugar
- Increasing artificial sweetener
- Replacing saturated fats with unsaturated fats
- Increasing fiber
- Limiting processed foods

Answer feedback:

Correct! All of the others are sustainable eating choices that are associated with improved health. Artificial sweeteners are associated with increased risk of stroke, heart disease, and death.

Next clue:

When fluid drains oh so foul, and the way of the kangaroo you have no time for now, use me to stent open the flow, and to the OB/GYN they will go. Fill me up with an 18-gauge needle; otherwise, the flow of pus will be feeble. But not too much, the balloon will explode. Find me for the next QR code.

Answer--Word catheter

Question 13:

Which of these is unlikely to help you achieve your physical wellbeing goals?

- Set both short- and long-term goals
- Set goals based on your priorities
- Use vague goals that are difficult to measure
- Work with a partner to hold each other accountable
- Write down your goals, both short- and long-term

Answer feedback:

Correct! Goals should be SMART--specific, measurable, achievable, realistic, and timely. Vague goals are difficult to use for motivation because they are hard to measure. All of the others are great ways to increase your likelihood of achieving your goals.

Next clue:

I am strong when what should be strong is broken. You won’t find me in the FED, but I am your only option at UH. Bro doctors say they can’t live without me.

Answer--plaster

Question 14:

What is one thing each group member will do to improve their quality of sleep? Each group member should have a different answer.

Answer feedback:

There are no incorrect answers! Potential options include ensuring seven hours of sleep at night, sleeping with blackout curtains, wearing sunglasses on the way home post night shift, or many others.

Next clue:

Here you will find the question for your victory to clinch, the hallway that leads to plastic for a pinch. From here candy abounds, and leaders for the department make their rounds.

Answer--nursing administrators’ office

Question 15:

Please list two changes that each of you can make in your food choices to improve your overall health. Each team member should list two separate changes.

Answer feedback:

There are no right or wrong answers here. Options include increased fruit and vegetable intake, decreased artificial sweeteners, increased water consumption, decreased red meat consumption, eating breakfast every day, or others.

Next clue:

Congratulations, you’ve reached the end of our game. Now back to the classroom for your prize to claim. The first team that makes it back with all answers complete wins a prize ever so sweet.

### Physical Wellbeing Scavenger Hunt Pearls

#### Overall Pearls

- To achieve change, set realistic short- and long-term goals, and write them down.^5^
- Work with a team to improve accountability.^5^
- Small changes to sleep, exercise, and nutrition habits add up to a big difference over time.^6^

#### Sleeping Pearls

- The average adult needs seven hours of sleep a night.^7^
  ○ Anything less creates a sleep debt, worsening health and physical and mental performance.^7^
  ○ Sleep debt is cumulative, and catch-up sleep (ie, sleeping in to make up the sleep debt) has not been shown to reverse the negative effects of sleep debt.^8^
- Zeitgebers, German for “time givers,” are environmental cues that synchronize our circadian rhythm.^9^
  ○ Things like sunlight, heat exposure, social interactions, and exercise can promote wakefulness, but can negatively affect the ability to sleep.^9^–^11^
  ○ Exercising more than two hours before going to bed can improve sleep quality and duration, but exercising within two hours of going to bed worsens quality of sleep.^10^
  ○ Consider sleeping in a cool, dark room with blackout curtains and minimizing screen exposure before bed.^9^,^11^
- Anchor sleep is maintaining a consistent four-hour block of sleep at the same time during a 24-hour period, which allows you to maintain your circadian rhythm and improve sleep quality.^12^
- Alcohol, which causes early wakening, and caffeine, which makes it difficult to initiate sleep, should be minimized before sleeping.^13^
- Transitioning shifts in a clockwise manner (eg, working a morning shift on day one and a swing shift on day two) is an easier transition than the opposite direction.^14^
- Brief naps before driving home improve safety when tired.^15^
  ○ Consider public transportation or the free taxi service if you have concerns or are questioning your ability to drive safely. There are no validated criteria to determine whether an individual is safe to drive, but subjective drowsiness, struggling to keep eyes open, swerving within a lane, and difficulty maintaining correct speed are all concerning for increased risk of a crash.^16^,^17^

#### Exercise Pearls

- The AHA recommends adults get 150 minutes of moderate aerobic activity or 75 minutes of vigorous aerobic activity spread throughout the week.^18^
  ○ Examples of moderate exercise include brisk walking, social dancing, Pilates, Tai Chi, and gardening.^18^
  ○ Examples of vigorous activity include hiking, aerobic dancing, running, swimming laps, weight training, high intensity interval training (HIIT), and heavy yardwork.^18^
- The AHA recommendations are great goals, but any amount of activity has been linked with decreased risk of chronic disease and improved quality of life.^18^
  ○ Set realistic goals. This will help long term success.^18^
  ○ Take the stairs or a brisk 10-minute walk a few times a day as a place to start.^18^

#### Nutrition Pearls

- Adding “healthy” foods to your diet is more sustainable than removing unhealthy items.^19^
  ○ Healthy options include fruits and vegetables, non-animal proteins, and unsaturated fats.^19^
    ▪ Increasing these type foods will help decrease sugar, limit processed foods, and limit saturated fats.^19^
  ○ Diet pills, crash dieting, and cleanses are not sustainable and could be dangerous.^19^
- Limit artificial sweeteners, which are associated with increased risks of heart attack and stroke, animal proteins, and ultra processed foods.^19^
